# The direct miR‐874‐3p‐target FAM84A promotes tumor development in papillary thyroid cancer

**DOI:** 10.1002/1878-0261.12941

**Published:** 2021-03-23

**Authors:** Yu Ding, Luyao Wu, Xi Zhuang, Jingsheng Cai, Houchao Tong, Yan Si, Hao Zhang, Xiaoting Wang, Meiping Shen

**Affiliations:** ^1^ Department of General Surgery the First Affiliated Hospital of Nanjing Medical University China

**Keywords:** FAM84A, miR‐874‐3p, papillary thyroid carcinoma, Wnt/β‐catenin signaling

## Abstract

With the improvement in diagnostic technology, the incidence of thyroid cancer (TC) is on the rise. Papillary thyroid carcinoma (PTC) is the most common pathological type of thyroid cancer; therefore, it is important to explore some valuable molecular targets to improve the treatment and prognosis of PTC. Studies have shown that family with sequence similarity 84, member A (FAM84A) is involved in the development of various tumors. However, the role of FAM84A in PTC remains unknown. Herein, we explored the biological function and specific molecular mechanism of FAM84A in PTC. Results indicated that FAM84A was upregulated in PTC tissues and cells. In addition, patients with higher FAM84A expression tended to possess larger tumor size, higher lymph node metastasis rate, and advanced TNM stage. Further studies indicated that downregulation of FAM84A could inhibit the development of PTC *in vitro* and *in vivo* by repressing the epithelial–mesenchymal transition (EMT) and Wnt/β‐catenin signaling pathway. Moreover, FAM84A was confirmed to be negatively regulated by tumor suppressor miR‐874‐3p. In conclusion, our findings suggest that FAM84A may act as a potential diagnostic and therapeutic target for PTC.

## Graphical abstract

This study describes the oncogenic role of FAM84A in papillary thyroid carcinoma (PTC). FAM84A promoted PTC cell proliferation and metastasis by activating the Wnt/β‐catenin signaling pathway and the EMT process. Additionally, the expression of FAM84A was suppressed by miR‐874‐3p. Altogether, our findings may provide potentially novel diagnostic and therapeutic targets in patients with PTC.

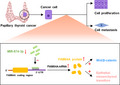

**Abbreviations**
3’‐UTR: 3’‐untranslated regions
CCK‐8: Cell Counting Kit‐8
EdU: 5‐ethynyl‐2’‐deoxyuridine
EMT: epithelial–mesenchymal transition
FAM84A: family with sequence similarity 84, member A
FBS: fetal bovine serum
GSEA: gene set enrichment analysis
IF: immunofluorescence
IHC: immunohistochemistry
SDS/PAGE: sodium dodecyl sulfate/polyacrylamide gel electrophoresis
TCGA: The Cancer Genome Atlas

## 1 Introduction

Thyroid cancer is one of the most common malignant tumors in the world, and its morbidity and mortality are relatively high among endocrine system tumors [1, 2]. Moreover, it is reported papillary thyroid carcinoma(PTC) accounts for 80–85% of all primary thyroid cancers [3]. Current treatment options for PTC include surgical resection, thyroid hormone suppression, and radioiodine therapy, which could bring a relatively good prognosis for PTC patients [4]. However, about 10% of patients are firstly diagnosed at the stage of local advanced or distant metastasis, which is the most common cause of thyroid cancer‐related death [5]. Therefore, it is urgent to explore the molecular mechanism of thyroid carcinogenesis and find new potential therapeutic targets.

Advancements in high‐throughput techniques such as The Cancer Genome Atlas (TCGA) project have favored development of data portals, providing large‐scale data of molecular aberrations at DNA, RNA, protein, and epigenetic levels in various types of tumors, and therefore helping the understanding of potential molecular mechanisms to carcinogenesis [6, 7, 8]. In this study, by screening TCGA database, the expression of family with sequence similarity 84, member A (FAM84A) was found upregulated in TC tissues compared with normal tissues. Previous study showed that FAM84A could promote the carcinogenesis of colon cancer by altering cell morphology and increased cell motility [9]. Another study indicated that FAM84A functioned as an oncogene to promote cell migration in mouse liver tumor [10]. Besides, the expression of FAM84A was also enhanced in other tumors such as bladder cancer and lung cancer [9]. However, the role of FAM84A in PTC has never been reported and remains completely unknown.

Gene set enrichment analysis (GSEA) is widely applied to explore gene interactions, which uses the Kolmogorov–Smirnov statistical variation to provide enrichment scores for each gene set [11]. In this study, according to the analysis of GSEA, we found that the Wnt/β‐catenin signaling pathway was enriched in groups with higher expression of FAM84A. Previous studies have shown that Wnt/β‐catenin signaling pathway plays a well‐established role in cell proliferation and tumor aggressiveness in TC [12, 13, 14]. However, the interaction between FAM84A and Wnt/β‐catenin signaling has not been confirmed in PTC yet.

Previous study indicated that FAM84A may be regulated in the post‐transcriptional level [9]. Notably, while the regulation of target genes by miRNAs is one of the most common ways through post‐transcriptional regulations [15], microRNAs are endogenous, highly conserved, small noncoding RNAs of approximately 17–25 nucleotides, mainly by binding to the 3’‐untranslated regions (3’‐UTR) of target mRNA and therefore causing its degradation or translational suppression [16, 17, 18]. It has been reported that miRNAs participated in various biological activities in cells such as proliferation, differentiation, apoptosis, metabolism, and stem cell maintenance [19, 20, 21]. However, the relationship between FAM84A and miRNAs remains unknown in PTC.

In this study, we elucidated the biological function, underlying mechanism and clinical significance of FAM84A in PTC. We observed that FAM84A was negatively regulated by miR‐874‐3p and played an oncogenic role through regulating Wnt/β‐catenin signaling in PTC. This work may provide insight into the development of PTC and highlight new potential targets for PTC treatment.

## 2 Materials and methods

### 2.1 Tissue specimens

Eighty pairs of human PTC and matched normal tissues were obtained from patients who underwent surgery at the First Affiliated Hospital of Nanjing Medical University (NMU). All of cases were diagnosed as PTC by at least two experienced pathologists. The tissues were stored at −80 °C until needed. Informed consent for the use of the tissues was obtained, and the Research Ethics Committee of NMU approved the study.

### 2.2 Cell culture

Human PTC cell lines (K‐1, TPC‐1, and IHH‐4) and a normal thyroid follicular epithelium cell line (Nthy‐ori3‐1) were obtained from the American Type Culture Collection (ATCC). Nthy‐ori3‐1 and K‐1 cells were cultured in RPMI‐1640 medium (Gibco, Carlsbad, CA, USA) with 10% fetal bovine serum (FBS). TPC‐1 cells were cultivated in DMEM high glucose (Gibco) supplemented with 15% FBS. A mixture of DMEM and RPMI‐1640 (1 : 1) supplemented with 10% FBS was used to maintain the IHH‐4 cell line. All media were added with 1% antibiotic solution (100 U·mL^−1^ penicillin and 100 mg·mL^−1^ streptomycin). The cultures were routinely grown at 37 °C in a humidified atmosphere containing 5% CO_2_.

### 2.3 RNA extraction and quantitative real‐time PCR

Total RNA was extracted from PTC samples and cells using the TRIzol reagent (Invitrogen, Carlsbad, CA, USA) according to the manufacturer’s protocol. The NanoDrop spectrophotometer (ND‐100, Thermo, Carlsbad, CA, USA) was adopted to measure the quality and concentration of RNA. Complementary DNA (cDNA) was synthesized from 1 μg of total RNA with the PrimeScript RT Master Mix Kit (TaKaRa, Kyoto, Japan). For miR‐874‐3p, the Hairpin‐it^TM^ miRNA qPCR Quantitation Kit (GenePharma, China) and SYBR Green Master Mix (Vazyme, Nanjing, China) were applied to perform reverse transcription and quantitative real‐time PCR, respectively, and its expression was detected by ABI StepOnePlus Real‐Time PCR System (Applied Biosystems, Foster City, CA, USA). The relative expression of FAM84A was normalized to GAPDH, while that of miR‐874‐3p was normalized to U6. All of the reactions were run in triplicate. Primer’s sequences were as follows: FAM84A forward 5’‐ATTCGGCTCGGGGTAGAG‐3’, reverse 5’‐TCTTCCTCATCATCCGAGAA‐3’; GAPDH forward 5'‐AGAAGGCTGGGGCTCATTTG‐3’, reverse 5'‐AGGGGCCATCCACAGTCTTC‐3’.

### 2.4 Cell transfection

The FAM84A short interfering RNA (si‐FAM84A), miRNA mimics, the short hairpin RNA targeting against FAM84A (sh‐FAM84A), and corresponding negative controls (NCs) were synthesized by GenePharma (Shanghai, China). Lipofectamine 3000 reagent (Invitrogen) was employed to facilitate transfection in TPC‐1 and K‐1 cells according to the manufacturer’s protocol. Stable cell lines were selected in cultures containing 3 µg·mL^−1^ puromycin for 10–15 days.

### 2.5 Western blot analysis

Protein was extracted and separated by sodium dodecyl sulfate/polyacrylamide gel electrophoresis (SDS/PAGE) and electrophoretically transferred to polyvinylidene fluoride (PVDF) membranes (Millipore, Darmstadt, Germany). After being blocked in 5% nonfat milk, the membranes were incubated with the following primary antibodies at 4 °C overnight: FAM84A (Abcam, 1 : 1500 Cambridge, MA, USA), E‐cadherin (CST, 1 : 1000), N‐cadherin (CST, 1 : 1000), vimentin (CST, 1 : 1000), CDK‐4 (CST, 1 : 1000), CDK‐6 (CST, 1 : 1000), Bcl‐2 (CST, 1 : 1000), Bax (CST, 1 : 1000), and β‐catenin (CST, 1 : 1000), followed by a brief wash and incubation with secondary antibody for 2 h at room temperature. Anti‐GAPDH and anti‐histone H3 antibodies were purchased from Beyotime (Shanghai, China) and used as loading control. The expression of protein was visualized by the enhanced chemiluminescence detection system.

### 2.6 Cell Counting Kit‐8 assay and colony formation assay

Cell viability was assessed with the Cell Counting Kit‐8 (CCK‐8; Dojindo, CK04‐500). After the desired treatment, cells (1*10^3^ cells/well) were seeded into 96‐well plates and incubated at 37 °C with 5% CO_2_. Absorbance was quantified at 450 nm using a spectrophotometer (Thermo Fisher, USA) after the transfected cells were incubated with CCK‐8 solutions for 2 h at 37 °C. The cell viability was monitored every 24 h.

Cell colony formation ability was measured by plate colony formation assay. Cells were seeded into 6‐well plates at 500 cells per well and cultivated in complete growth media for about 2 weeks. Then, the number of colonies was counted manually after the colonies fixed with methanol and stained with 0.1% crystal violet (Beyotime, Shanghai, China) in PBS for 15 min.

### 2.7 5‐Ethynyl‐2’‐deoxyuridine assay

The 5‐Ethynyl‐2’‐deoxyuridine (EdU) assay kit (RiboBio, Guangzhou, China) was employed to evaluate cell growth ability. The transfected cells, cultured in a well of a 96‐well plate at a density of 5000 cells per well, were labeled with 50 μm of EdU and incubated for additional 2 h before being fixed with 4% paraformaldehyde (pH = 7.4) for 30 min. Then, 0.5% Triton X‐100 was added to each well and incubated at room temperature for 10 min. After that, anti‐EdU working solution (Apollo^R^ reaction cocktail) and Hoechst 33342 were successively employed to stain the cells. The EdU‐labeled root cells were observed under a fluorescence microscope (Nikon, Tokyo, Japan).

### 2.8 Wound‐healing assay and transwell assay

For the wound‐healing assays, TPC‐1 and K‐1 cells treated differently were plated in 6‐well plates and cultured until reaching confluency. Wounds were made in the monolayer of the cells using a standard 200‐μL pipette tip. Culture medium was replaced with serum‐free medium. Images were taken at 0 h and 24 h after wounding. The wound area was measured, and the percentage of wound healing was estimated by using the imagej software (NIH, Bethesda, MD, USA).

Cell migration and invasion was measured in transwell chambers with 8 μm pores (Corning, New York, NY, USA). For the cell migration assays, treated cells (2*10^4^ cells/well) suspended in 200 μL serum‐free medium were added onto the upper chambers, and the lower chambers were supplemented with 500 μL complete culture medium as a chemoattractant. After incubation for 24 h, cells on the upper chambers were mechanically removed, and those that had penetrated the lower surface were fixed, stained, and photographed. For the invasion assays, cells were seeded on the upper chambers precoated with Matrigel (BD Bioscience, Franklin Lakes, NJ, USA), other steps were exactly the same as the migration assays.

### 2.9 Flow cytometric analysis

Apoptosis was measured by the flow cytometric detection (FACScan, BD Biosciences) of phosphatidylserine externalization using Annexin V‐APC/PI Apoptosis Detection Kit (Multiscience, Hangzhou, China). Cells were discriminated into viable cells, dead cells, early apoptotic cells, and late apoptotic cells, and then, the relative ratio of early and late apoptotic cells accounting for total cells was compared from each treated group.

Cells for cell cycle analysis were stained with propidium iodide staining solution (Multisciences, Hangzhou, China) following the protocol and analyzed by the flow cytometer mentioned above. The percentage of the cells in G0‐G1, S, and G2‐M phases were counted and compared.

### 2.10 TOP‐flash/FOP‐flash luciferase reporter assay

Cells treated differently were cotransfected with TOP/FOP‐flash expression plasmid and pRL‐TK using Lipofectamine 3000. Twenty‐four hours later, the luciferase activity was measured with a dual‐luciferase reporter kit (Promega, Madison, WI, USA). The relative ratio of firefly luciferase activity to Renilla luciferase activity was determined as the TOP‐flash reporter activity.

### 2.11 Luciferase reporter assay

Cells were cultured in 24‐well plates and cotransfected with a Pgl3 luciferase reporter vector (Promega), containing the 3’‐UTR (wild‐type or mutant) of FAM84A and miR‐874‐3p mimics using Lipofectamine 3000 (Invitrogen). After 48 h of transfection, luciferase activity was measured using a dual‐luciferase kit (Promega) in accordance with the manufacturer’s protocol.

### 2.12 RNA immunoprecipitation (RIP) and RNA pull‐down assay

Cells treated differently were lysed with RIP buffer, which is from the Magna RNA immunoprecipitation kit (Millipore), and the magnetic beads conjugated with anti‐IgG antibody or anti‐Ago2 antibody were incubated with the above cell lysis buffer at 4 °C. Finally, the elution of RIP‐Ago2 and RIP‐IgG was extracted, followed by qRT‐PCR.

Cells treated with biotinylated‐miR‐874‐3p (Bio‐miR‐874‐3p) or biotinylated‐miR‐NC (Bio‐NC) from GenePharma (Shanghai, China) were harvested and lysed, and then, the streptavidin‐coated magnetic beads (Invitrogen) were incubated with the cell lysis. After washing, magnetic bead–RNA–protein complex was collected, followed by total RNA isolation and qRT‐PCR.

### 2.13 Animal experiments

Twenty 4‐week‐old female BALB/c nude mice were purchased from the animal center of NMU. For each mouse, 1*10^7^ stably transfected cells were inoculated subcutaneously into the same side armpit (5 mice for each group) in 100 μL phosphate‐buffered saline (PBS). The tumor volume was assessed by measuring the 2 perpendicular dimensions using a caliper and the formula: *V* = 0.5 × *D* × *d* ^2^ (*V*, volume; *D*, longitudinal diameter; *d*, latitudinal diameter). Twenty‐five days after inoculation, we euthanized the mice, excised the implanted tumor, and examined tumor subcutaneous growth. All of the above animal procedures were approved by the Nanjing Medical University Institutional Animal Care and Use Committee.

### 2.14 Immunohistochemistry (IHC)

Immunohistochemistry staining was performed on tumor sections from PTC patients and nude mice. The sections were treated with primary antibodies against FAM84A (1 : 200, Abcam) or Ki‐67 (1 : 500, Abcam), which was used to evaluate the proliferation, and then incubated with secondary antibodies. The images of all samples were observed with a microscope.

### 2.15 Immunofluorescence (IF)

Cells treated differently were fixed in formaldehyde for 20 min and permeabilized with Triton X‐100 for 5 min at room temperature. Then blocked for 1 h with 2% BSA, cells were incubated with primary antibodies β‐catenin (1 : 100, CST) and FAM84A (1 : 200, Abcam) at 4 °C overnight. Afterward, cells were incubated with secondary antibodies (Cy3™ goat anti‐rabbit IgG, Jackson ImmunoResearch, West Grove, PA, USA) for 1 h at room temperature. The cell nuclei were stained with DAPI for 5 min. Photographs were taken using a confocal microscope (Nikon).

### 2.16 Public data analysis

Gene expression data from TCGA dataset were downloaded from the UCSC cancer browser (https://xenabrowser.net/datapages/). In addition, the GSE66783 data were obtained from NCBI/GEO (https://www.ncbi.nlm.nih.gov/geo/). The gene set enrichment analysis (GSEA) software was downloaded for use from the Broad Institute Website (http://software.broadinstitute.org/gsea/index.jsp). If the p value was less than 0.05 and the false discovery rate (FDR) was less than 0.25, the findings were considered statistically significant(https://www.gseamsigdb.org/gsea/doc/GSEAUserGuideFrame.html?_Interpreting_GSEA_Results). TargetScan, miRanda, RNAhybrid, and miRWalk (http://zmf.umm.uni‐heidelberg.de/apps/zmf/mirwalk/predictedmirnagene.html) were used to analyze the miRNA‐mRNA relationships.

### 2.17 Statistical analysis

Data are presented as mean ± standard deviations. All statistical analyses were carried out with Statistical Product and Service Solutions (spss) software version 25.0 (SPSS Inc., Chicago, IL, USA) or graphpad prism software version 6.0 (San Diego, CA, USA). Clinicopathological results were compared using the chi‐square detection. Spearman’s correlation analysis was conducted to analyze the correlation between Ki‐67, β‐catenin, miR‐874‐3p, and FAM84A. Student’s t‐test and ANOVA test were used to compare the treated group and control group. *P* < 0.05 was considered statistically significant (*P < 0.05, **P < 0.01, and ***P < 0.001).

## 3 Results

### 3.1 FAM84A is upregulated in human PTC samples and cell lines

**Fig. 1:**
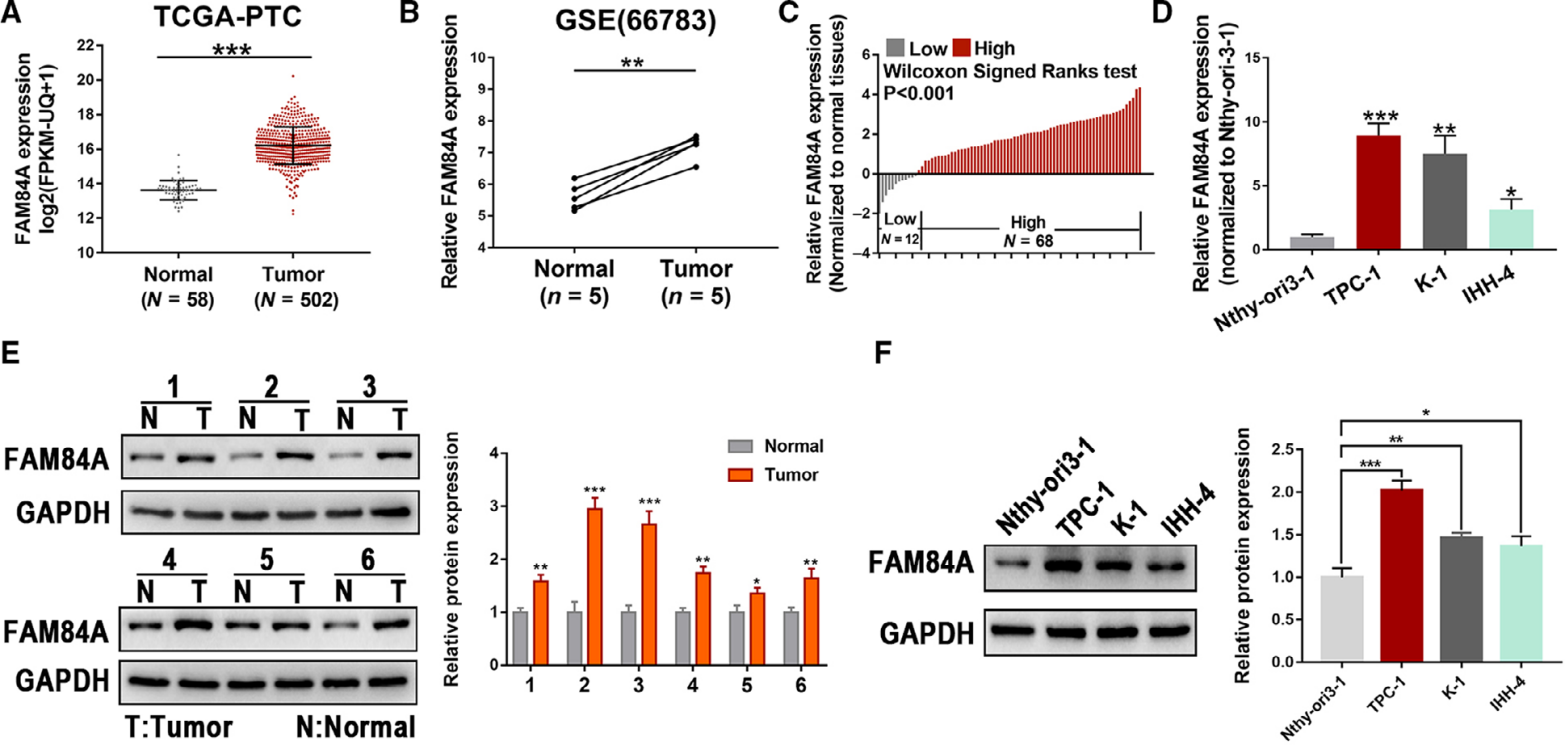
Expression of FAM84A in PTC tissues and cells. (A, B) Expression of FAM84A was compared in unpaired PTC tissues from TCGA database and paired PTC tissues from GEO database, which showed that FAM84A was upregulated in TC. (C) FAM84A was observed upregulated in 80 paired PTC tissues and adjacent normal tissue. (D) The expression of FAM84A mRNA was upregulated in PTC cell lines than that in normal thyroid epithelial cell line Nthy‐ori3‐1. (E) Protein expression from 6 randomly selected PTC tissues showed FAM84A was upregulated. (F) FAM84A was observed upregulated in PTC cells than that in normal thyroid epithelial cell line at protein level. Results were representative of three independent experiments and presented as the mean ± SD, two‐tailed *t*‐tests, *P < 0.05, **P < 0.01, and ***P < 0.001.

By analyzing public databases TCGA and GSE66783, FAM84A was identified as possibly associated with thyroid tumorigenesis, considering the high expression of FAM84A in TC samples than in normal tissues (Fig. 1A,B). Then, we detected the expression of FAM84A in our own 80 paired PTC tissues and cell lines by qRT‐PCR, which indicated that the expression of FAM84A was significantly upregulated (Fig. 1C,D). Meanwhile, 6 paired PTC tissues were selected randomly to detect the FAM84A protein expression, which showed a higher FAM84A protein level in PTC tissues (Fig. 1E). Similar results were observed in PTC cell lines (Fig. 1F). We also detected the protein level of FAM84A with those 80 pairs of samples by IHC, the results were consistent with previous findings (Fig. S1a and b). Meanwhile, the correlation between the clinicopathologic features of 80 PTC patients and FAM84A expression was analyzed, which displayed that larger tumor size, higher lymph node metastasis rate and advanced TNM stage were remarkably associated with higher FAM84A expression (Table S1). These results revealed that FAM84A was upregulated in PTC and may play a key role in the development of PTC.

### 3.2 Knockdown of FAM84A inhibits the proliferation of PTC cells in vitro

**Fig. 2:**
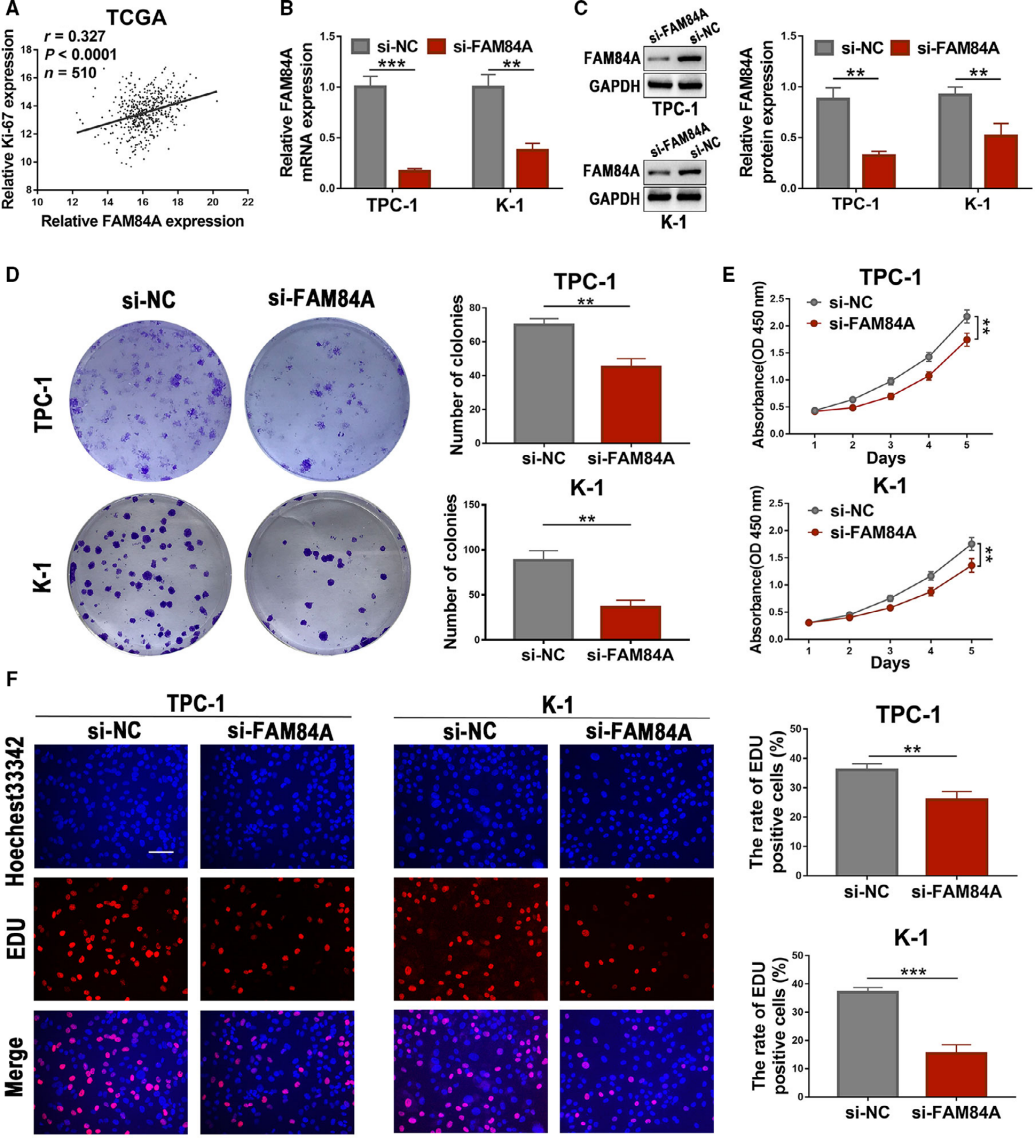
Effects of FAM84A on proliferation and growth of PTC cells. (A) According to the data originated from TCGA database, a positive correlation was observed between the expression of FAM84A and proliferation marker Ki‐67 in TC. (B, C) After knockdown of FAM84A with si‐FAM84A or si‐NC in TPC‐1 and K‐1 cells, the mRNA and protein expressions of FAM84A were identified downregulated by qRT‐PCR and western blot. (D–F) Colony formation assay, CCK‐8 assay, and EdU assay showed that the proliferation ability of TPC‐1 and K‐1 cells suppressed after knockdown of FAM84A. Scale bar = 200 μm. Results were representative of three independent experiments and presented as the mean ± SD, Spearman’s correlation analysis for A; two‐tailed *t*‐tests for B, C, D, And F; and ANOVA test for E, **P < 0.01 and ***P < 0.001.

Ki‐67 is a proliferative cell‐associated antigen, which is widely used to evaluate the proliferation ability of tumor cells [22]. Thus, the correlation between FAM84A expression and Ki‐67 was analyzed by utilizing TCGA database, and a positive correlation was observed (Fig. 2A). In purpose of investigating the biological functions of FAM84A in PTC, TPC‐1 and K‐1 cell lines with relatively higher FAM84A expression were selected for the further experiments. We knocked down FAM84A expression by transfecting with siRNAs in TPC‐1 and K‐1 cells, the efficiency of transfection was verified by qRT‐PCR and western blot (Fig. 2B,C). Colony formation assay indicated that downregulation of FAM84A significantly reduced the cell colony ability of TPC‐1 and K‐1 cells (Fig. 2D). Furthermore, the CCK‐8 assay and the EdU assay indicated that downregulation of FAM84A could attenuate the cell growth ability and the DNA synthesis (Fig. 2E,F). These results suggested that downregulation of FAM84A inhibited the proliferation of PTC cells *in vitro*.

### 3.3 Knockdown of FAM84A induces cell cycle arrest and apoptosis, and inhibits tumorigenesis in vivo

**Fig. 3:**
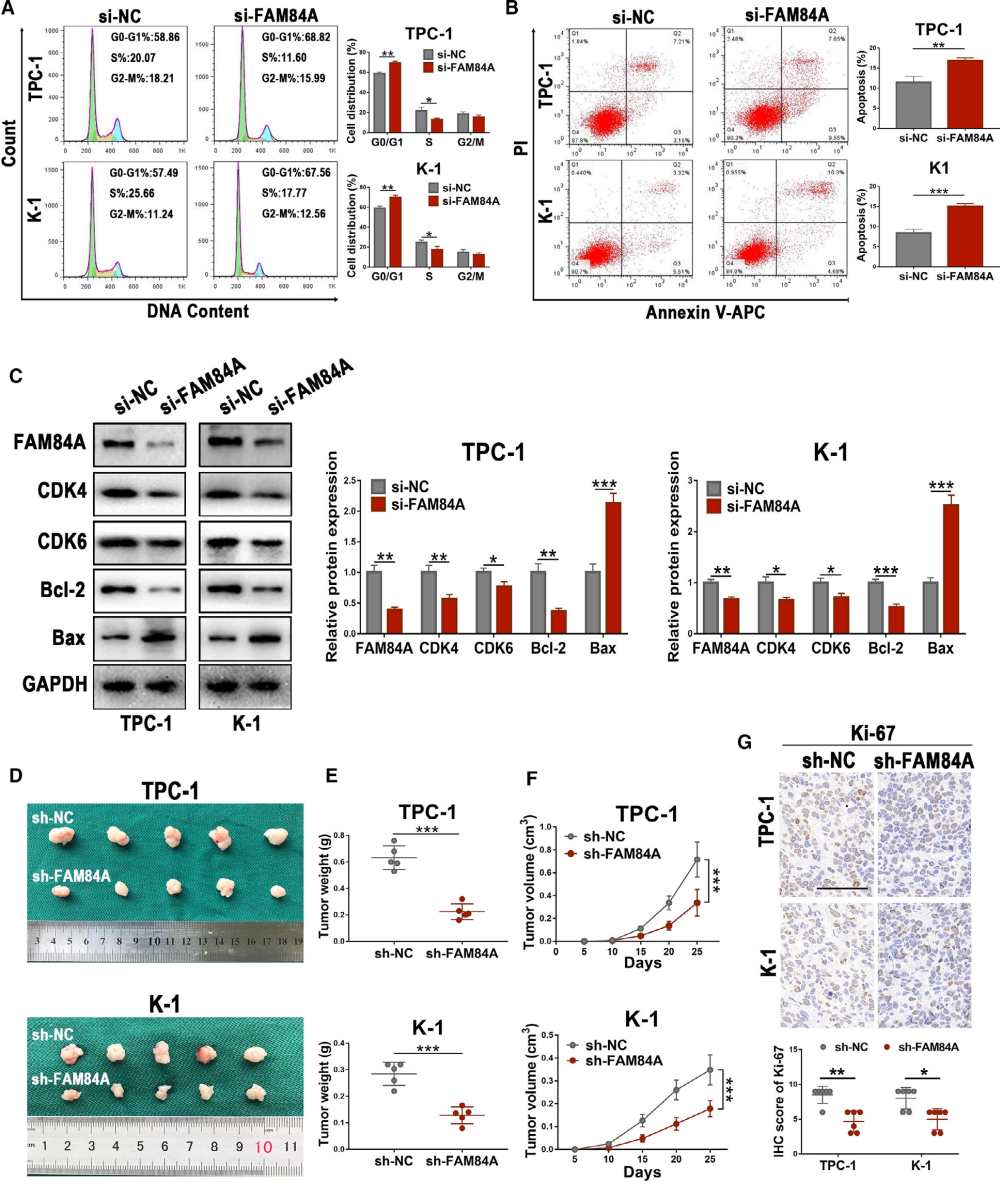
Effects of FAM84A on PTC cell cycle, apoptosis in vitro and tumorigenicity in vivo. (A) Flow cytometric analyses of TPC‐1 and K‐1 cells revealed that knockdown of FAM84A induced cell cycle arrest in the G0/G1 phase and the number of cells in the S phase was reduced. (B) PTC cells treated with si‐FAM84A possessed a higher apoptotic rate in comparison with the negative control groups. (C) Western blot assays showed that cell cycle‐related proteins, CDK4 and CDK6, were significantly reduced after downregulation of FAM84A. Furthermore, the apoptosis‐related protein Bax was increased and the anti‐apoptotic protein Bcl‐2 was decreased in TPC‐1 and K‐1 cells treated with si‐FAM84A. (D) Tumors harvested from the different groups of nude mice treated with different cells that transfected with sh‐FAM84A or sh‐NC, respectively. (E, F) The size and weight of tumor explants harvested from sh‐FAM84A groups were relatively smaller than those generated from their control groups. (G) Proliferation marker Ki‐67 expression in tumor tissues from nude mice treated differently was measured by IHC, which suggested that downregulation of FAM84A inhibited the expression of Ki‐67 in xenografts. Scale bar = 50 μm. Results were representative of three independent experiments and presented as the mean ± SD, ANOVA test for (F); and two‐tailed *t*‐tests for the others, *P < 0.05, **P < 0.01, and ***P < 0.001.

The cell apoptosis and cell cycle arrest are important factors that could influence PTC cell proliferation. Flow cytometric analysis of TPC‐1 and K‐1 cells revealed that knockdown of FAM84A induced cell cycle arrest at G0/G1 phase (Fig. 3A). Meanwhile, these two PTC cells treated with si‐FAM84A possessed a higher apoptotic rate in comparison with the NC group (Fig. 3B). Next, western blot showed that G1/S phase‐related proteins, CDK4 and CDK6, were significantly reduced after downregulation of FAM84A. Furthermore, the anti‐apoptotic protein Bcl‐2 was decreased and the apoptosis‐related protein Bax was increased in TPC‐1 and K‐1 cells transfected with si‐FAM84A (Fig. 3C). To determine whether knockdown of FAM84A could regulate tumor growth in vivo, TPC‐1 and K‐1 cells transfected with sh‐FAM84A or their negative control were inoculated subcutaneously into the flanks of nude mice. We discovered that the size and weight of tumor explants harvested from sh‐FAM84A groups were relatively smaller than those generated from their control group (Fig. 3D‐F). Meanwhile, proliferation marker Ki‐67 of tumor explants was detected by IHC staining, it suggested that downregulation of FAM84A inhibited the expression of Ki‐67 in xenografts (Fig. 3G). Herein, these results displayed that knockdown of FAM84A could induce G1 phase cell cycle arrest and PTC cell apoptosis, and inhibit tumorigenesis in vivo.

### 3.4 Knockdown of FAM84A suppresses migration and invasion of PTC cells

**Fig. 4:**
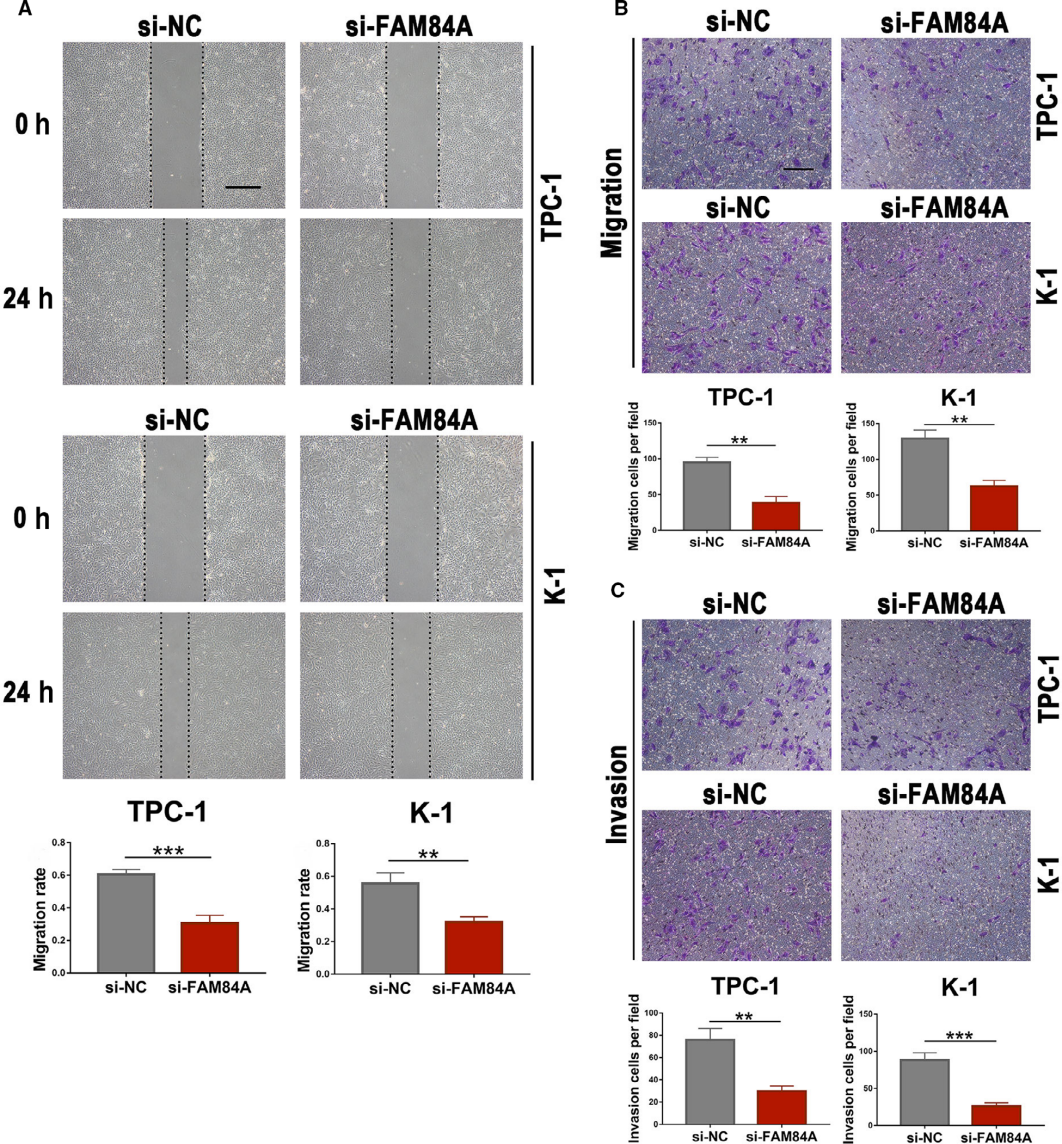
Effects of FAM84A on migration and invasion of PTC cells. (A) Wound‐healing assay was used to detect the migration ability of TPC‐1 and K‐1 cells transfected with si‐FAM84A or si‐NC, which suggested that the wound closure of PTC cells after transfection with si‐FAM84A was impaired compared with the negative control cells. Scale bar = 100 μm. (B, C) Transwell assay was used to detect the migration and invasion ability of PTC cells after knockdown of FAM84A, which revealed that knockdown of FAM84A significantly suppressed the migration and invasion ability of PTC cells. Scale bar = 200 μm. Results were representative of three independent experiments and presented as the mean ± SD, Two‐tailed *t*‐tests, **P < 0.01 and ***P < 0.001.

Transwell assays and the wound‐healing experiments were utilized to explore whether FAM84A could influence PTC cell migration and invasion. The wound‐healing assays suggested that the migration ability of K‐1 and TPC‐1 cells after transfected with si‐FAM84A was dramatically impaired compared with the control cells (Fig. 4A). Transwell assays without Matrigel demonstrated that knockdown of FAM84A suppressed K‐1 and TPC‐1 cell motility compared with the control groups (Fig. 4B), while transwell assays with Matrigel showed that these two PTC cell lines presented with significantly reduced invasiveness ability after knockdown of FAM84A (Fig. 4C). Collectively, these results indicated that knockdown of FAM84A significantly suppressed the migration and invasion ability of PTC cells *in vitro*.

### 3.5 FAM84A knockdown may inhibit the EMT characteristics and Wnt/β‐catenin signaling pathway in PTC

**Fig. 5:**
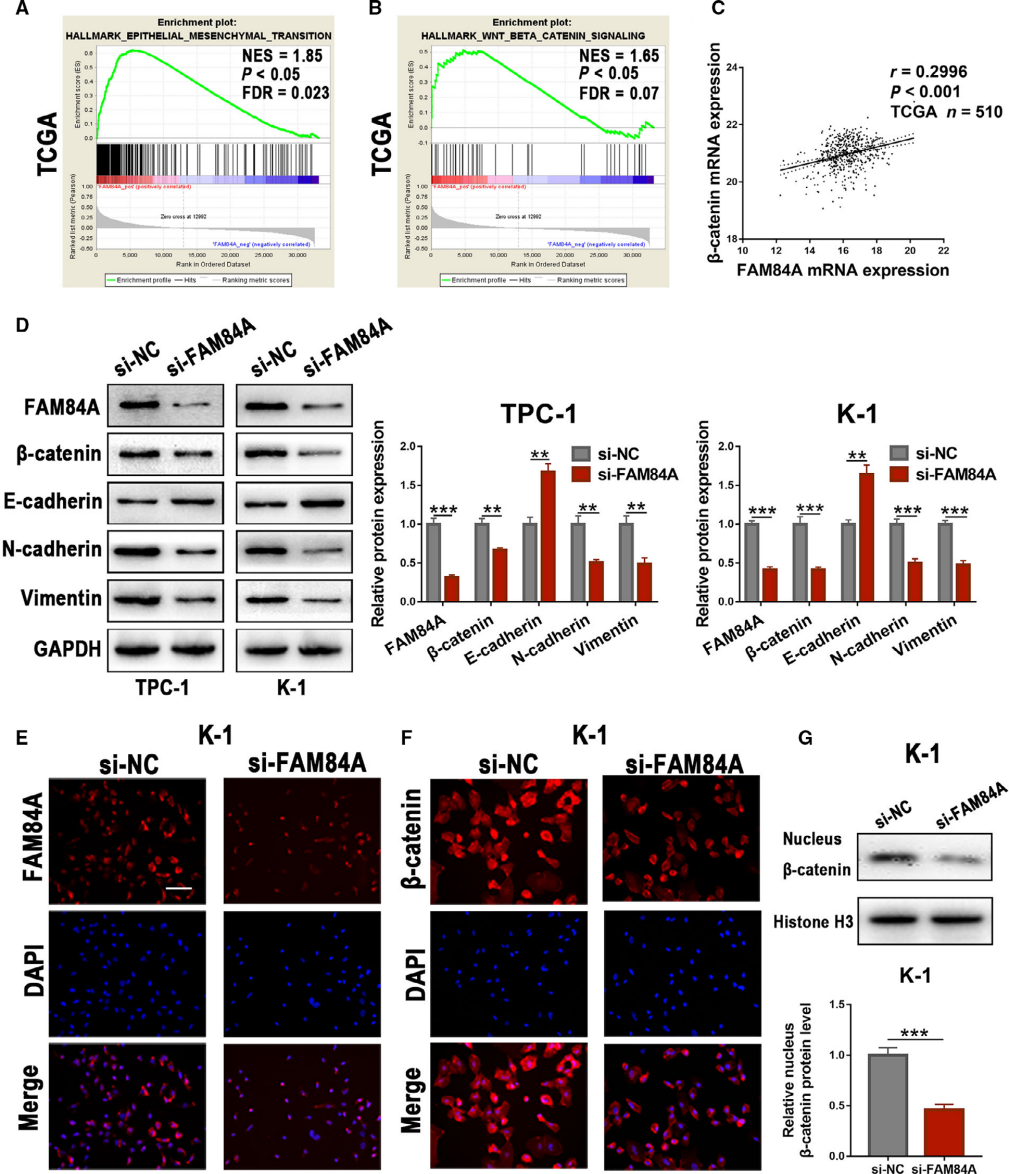
Effects of FAM84A on EMT and Wnt/β‐catenin signaling pathway in PTC. (A, B) Based on TCGA database, GSEA was performed to identify gene sets and pathways correlated with FAM84A, which showed that high FAM84A expression was associated with the activity of EMT and Wnt/β‐catenin signaling pathway. (C) A positive correlation was observed between FAM84A expression and β‐catenin expression by analyzing TCGA database. (D) Knockdown of FAM84A in TPC‐1 and K‐1 cell lines decreased the protein expression of vimentin and N‐cadherin, but increased the protein expression of E‐cadherin. Additionally, a decrease in β‐catenin was observed in si‐FAM84A groups compared with the negative control groups. (E, F) Immunofluorescence analysis indicated that FAM84A was mainly located in cytoplasm, β‐catenin was located both in cytoplasm and in nuclei, and that downregulation of FAM84A inhibited expression of nuclear β‐catenin. Scale bar = 200 μm. (G) Western blot further revealed that downregulation of FAM84A inhibited the protein expression of nucleus β‐catenin. Results were representative of three independent experiments and presented as the mean ± SD, Spearman’ s correlation analysis for **c**; and two‐tailed *t*‐tests for the others, **P < 0.01 and ***P < 0.001.

Gene set enrichment analysis (GSEA) is a powerful method of analyzing and interpreting microarray using biological knowledge, in which the expression of each gene on the list is weighted based on its log fold change [23]. In order to identify gene sets and pathways correlated with FAM84A, TCGA mRNA data were utilized to perform GSEA. Results from GSEA showed that FAM84A expression was positively associated with Wnt/β‐catenin signaling pathway and epithelial–mesenchymal transition (Fig. 5A,B). Meanwhile, we discovered that there was a positive correlation between FAM84A expression and β‐catenin expression by analyzing TCGA database (Fig. 5C). Then, protein of N‐cadherin, vimentin, and E‐cadherin that associated with EMT markers and protein of β‐catenin that associated with Wnt/β‐catenin signaling pathway were detected. We found that knockdown of FAM84A in TPC‐1 and K‐1 cell lines decreased the protein expression of vimentin and N‐cadherin, but increased the protein expression of E‐cadherin. Additionally, a decrease in β‐catenin was observed in si‐FAM84A groups compared with the NC groups (Fig. 5D). Meanwhile, a remarkable epithelial‐like shape morphological change was found in TPC‐1 and K‐1 cells transfected with FAM84A targeting siRNA (Fig. S2a). To further explore the role of FAM84A in Wnt/β‐catenin signaling pathway, immunofluorescence analysis was performed, which indicated that downregulation of FAM84A inhibited the expression of nucleus β‐catenin (Fig. 5E,F, Fig. S3a and b). Western blot analysis further confirmed that knockdown of FAM84A inhibited the protein expression of nucleus β‐catenin (Fig. 5G, Fig. S3c). Moreover, TOP/FOP transcriptional activity was remarkably inhibited in PTC cells treated with si‐FAM84A (Fig. S2b). Thus, these results preliminarily revealed that FAM84A knockdown may inhibit the epithelial–mesenchymal transition and Wnt/β‐catenin signaling pathway in PTC.

### 3.6 FAM84A is a direct downstream target of miR‐874‐3p

**Fig. 6:**
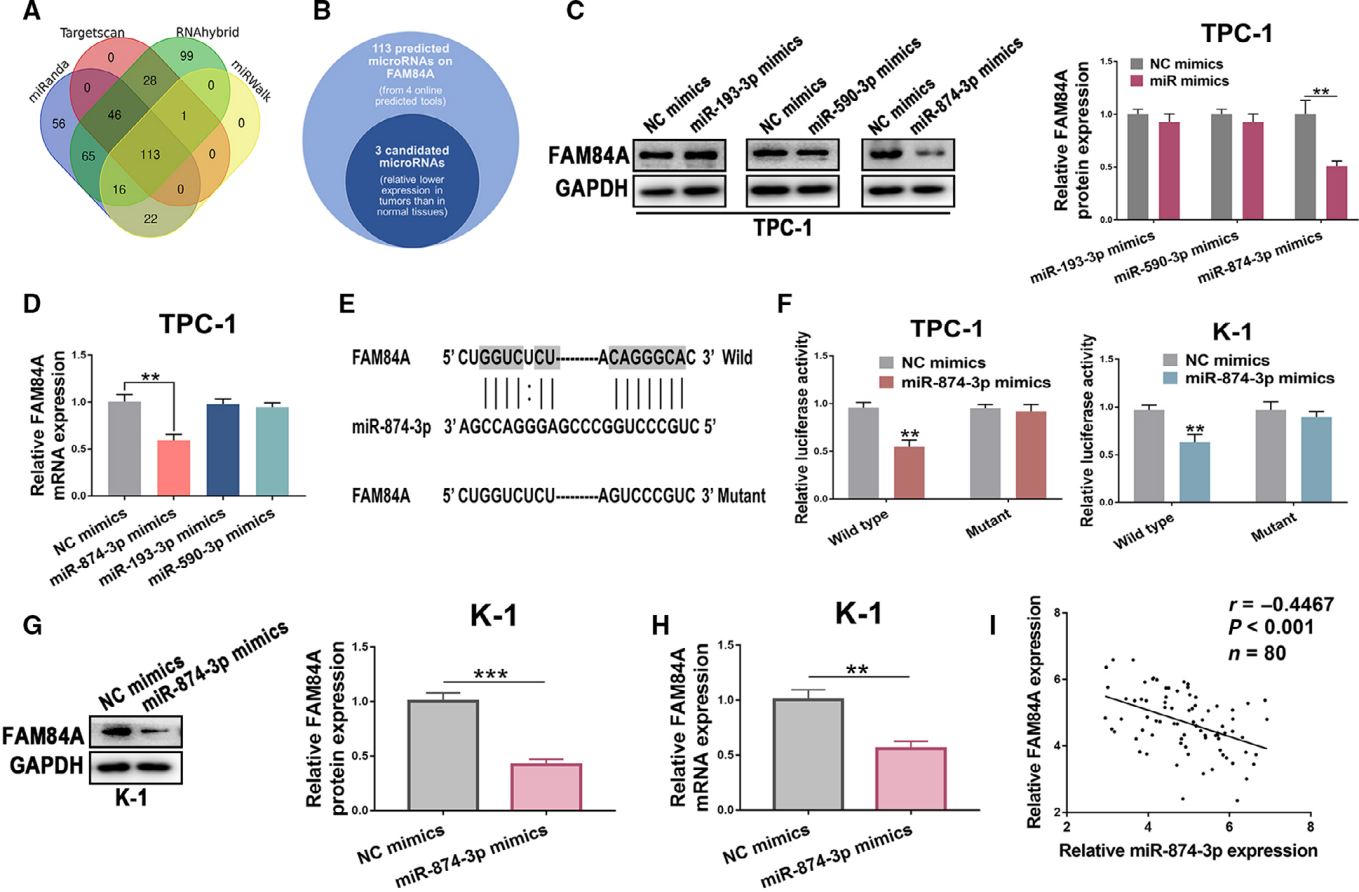
The regulatory effects of FAM84A in PTC is mediated directly by miR‐874‐3p. (A, B) MiRanda, TargetScan, RNAhybrid, and miRWalk were used to predict related miRNAs binding to sequence of FAM84A 3'UTR, and 113 miRNAs were found, 3 miRNAs were selected for they displayed remarkably lower expression in TC samples from TCGA datasets. (C, D) miR‐874‐3p, miR‐193‐3p, and miR‐590‐3p were upregulated to detect the FAM84A protein and mRNA expression in TPC‐1 cells, which showed that when miR‐874‐3p was upregulated, FAM84A was the most changed one at protein and mRNA expression level. (E) Predicted sequence that miR‐874‐3p may bind to FAM84A 3'UTR region. (F) miR‐874‐3p‐mimics prominently reduced luciferase activity in FAM84A‐wild not in FAM84A‐mut in K‐1 and TPC‐1 cells. (G, H) The protein and mRNA expressions of FAM84A were decreased after K‐1 cells transfected with miR‐874‐3p‐mimics. (I) A negative correlation was observed between FAM84A expression and miR‐874‐3 expression by analyzing our own 80 PTC tissues. Results were representative of three independent experiments and presented as the mean ± SD, Spearman’ s correlation analysis for **i**; and two‐tailed t‐tests for the others, **P < 0.01 and ***P < 0.001.

In order to explore the upstream targets of FAM84A in PTC, miRNAs that might regulate FAM84A were screened and predicted by using four bioinformatic websites including miRanda, TargetScan, RNAhybrid, and miRWalk, and 113 miRNAs were found (Fig. 6A). Among these predicted miRNAs, miR‐874‐3p, miR‐193‐3p, and miR‐590‐3p were selected for further verification as they acquired consistently high score in these bioinformatic websites and displayed remarkably lower expression in TC samples from TCGA datasets (Fig. 6B). Subsequently, these three candidate miRNAs were upregulated, and then protein and mRNA expression levels of FAM84A were detected in TPC‐1 cells. The results indicated that upregulation of miR‐874‐3p was accompanied by most downregulated expression of FAM84A(Fig. 6C,D). Then, the dual‐luciferase reporter assay showed that overexpression of miR‐874‐3p significantly reduced the relative luciferase activity of the wild‐type FAM84A (Fig. 6E,F). Meanwhile, results from RNA immunoprecipitation assay and RNA pull‐down assay showed that FAM84A level was abundantly enriched in RIP‐Ago2 (Fig. S4a and b) or bio‐miR‐874‐3p (Fig. S4c and d) in TPC‐1 and K‐1 cells transfected with miR‐874‐3p mimic. We continued to explore the effect of upregulation of miR‐874‐3p on FAM84A in K‐1 cells, the results were consistent with what we found in TPC‐1 cells (Fig. 6G,H). Additionally, the FAM84A was found inversely correlated with miR‐874‐3p expression in our own 80 PTC tissues (Fig. 6I). These results revealed that FAM84A expression was mediated by the regulation of miR‐874‐3p, at least in part.

### 3.7 miR‐874‐3p is downregulated and is a tumor‐suppressing miRNA in PTC

**Fig. 7:**
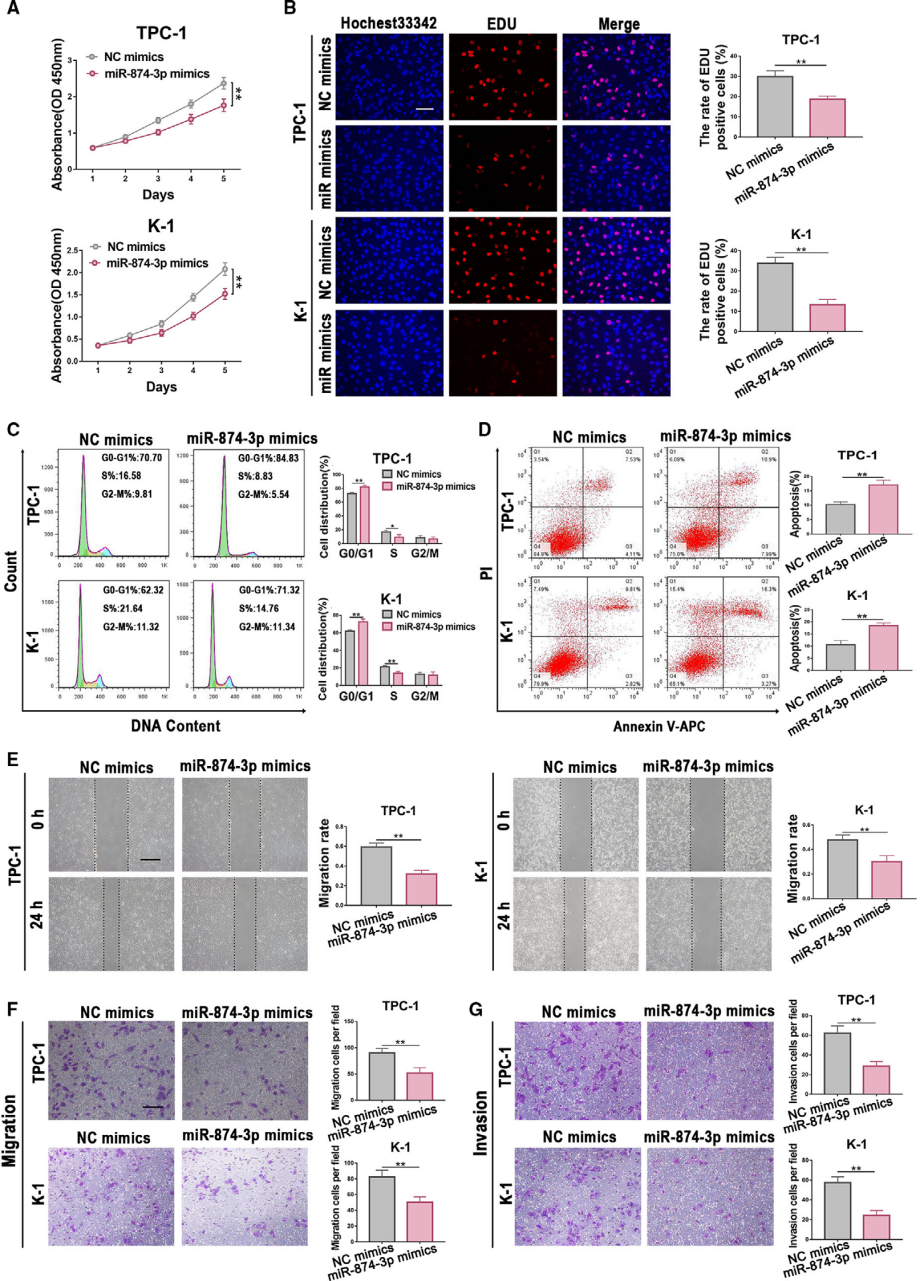
miR‐874‐3p acts as a tumor suppressor in PTC. (A, B) From CCK‐8 assay and EdU assay, we found that overexpression of miR‐874‐3p attenuated the proliferation of PTC cells. Scale bar = 200 μm. (C, D) Flow cytometry analysis suggested that upregulation of miR‐874‐3p could increase the number of cells arrested in the G0/G1 phase and induce cell apoptotic. (E–G) From wound‐healing assay and transwell assay, the migration and invasion ability of TPC‐1 and K‐1 cells decreased after overexpressing miR‐874‐3p. For wound‐healing assay, scale bar = 100 μm. For transwell assay, scale bar = 200 μm. Results were representative of three independent experiments and presented as the mean ± SD, ANOVA test for (A); and two‐tailed t‐tests for the others, *P < 0.05 and **P < 0.01.

According to data from TCGA database and our own 80 paired PTC tissues, we discovered that the expression of miR‐874‐3p was declined in PTC (Fig. S5a and b). Besides, miR‐874‐3p was also downregulated in TPC‐1 and K‐1 cells (Fig. S5c). To investigate the biological functions of miR‐874‐3p in PTC, we transfected TPC‐1 and K‐1 cells with miR‐874‐3p mimics and their negative control groups (Fig. S5d). By conducting CCK‐8 assay and EdU assay, we found that overexpression of miR‐874‐3p attenuated the proliferation of PTC cells (Fig. 7A,B). Flow cytometry analysis suggested that overexpression of miR‐874‐3p could increase the number of cells arrested in the G0/G1 phase and induce cell apoptosis (Fig. 7C,D). From transwell and wound‐healing assays, we found that upregulation of miR‐874‐3p decreased the migration and invasion ability of TPC‐1 and K‐1 cells (Fig. 7E–G). These results revealed that miR‐874‐3p was a tumor‐suppressing miRNA that inhibited cell proliferation, migration, and invasion in PTC.

### 3.8 The regulation of FAM84A on PTC cells is mediated by miR‐874‐3p

**Fig. 8:**
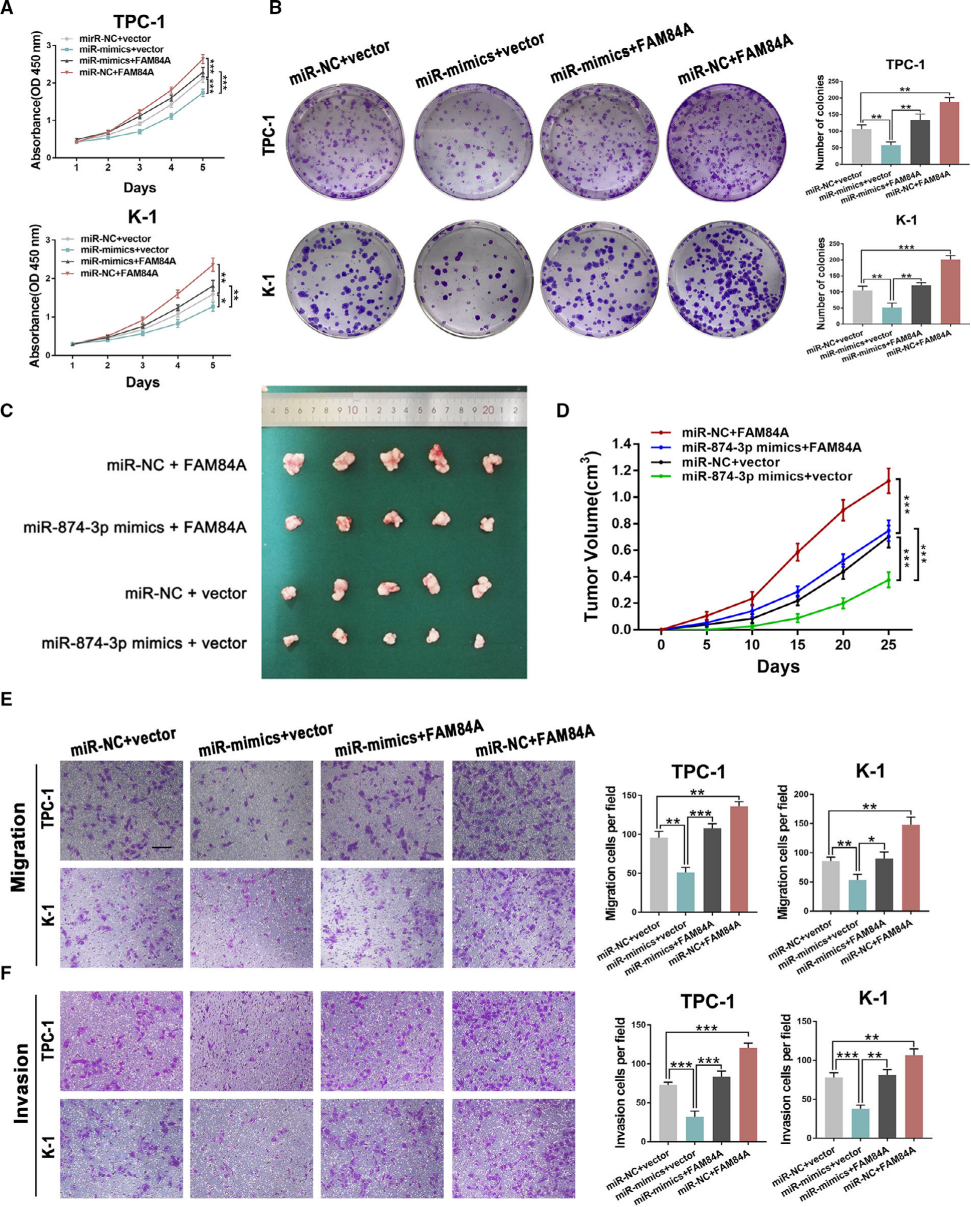
FAM84A could partly counteract the effect of miR‐874‐3p in PTC. (A–D) CCK‐8 assay, colony formation assay, and tumor formation assay displayed that miR‐874‐3p‐mediated suppression of PTC cell growth could be reversed by upregulation of FAM84A. (E, F) The effect on PTC cell migration and invasion was evaluated by transwell assays as indicated. Scale bar = 200 μm. Results were representative of three independent experiments and presented as the mean ± SD, ANOVA test for A, C, and D; and two‐tailed t‐tests for B, E, And F, *P < 0.05, **P < 0.01, and ***P < 0.001.

To further explore the biological interactions between FAM84A and miR‐874‐3p in PTC, TPC‐1 and K‐1 cells were cotransfected with miR‐874‐3p‐mimics, FAM84A vector, and corresponding negative control accordingly. Through conducting CCK‐8 assay, colony formation assay, and EdU assay, it displayed that suppressed ability of cell proliferation by miR‐874‐3p could be reversed by upregulation of FAM84A (Fig. 8A,B; Fig. S6a). Moreover, the results of rescue experiments in nude mice were consistent with what we found in vitro (Fig. 8C,D). Meanwhile, an analogous mode was also detected via conducting transwell assay and wound‐healing assay in which miR‐874‐3p‐mediated inhibition of cell migration and invasion was nullified by FAM84A vector (Fig. 8E,F; Fig. S6b). Taken together, these results revealed that FAM84A was a vital downstream target of miR‐874‐3p.

### 3.9 miR‐874‐3p is involved in EMT and Wnt/β‐catenin signaling pathway by targeting FAM84A

**Fig. 9:**
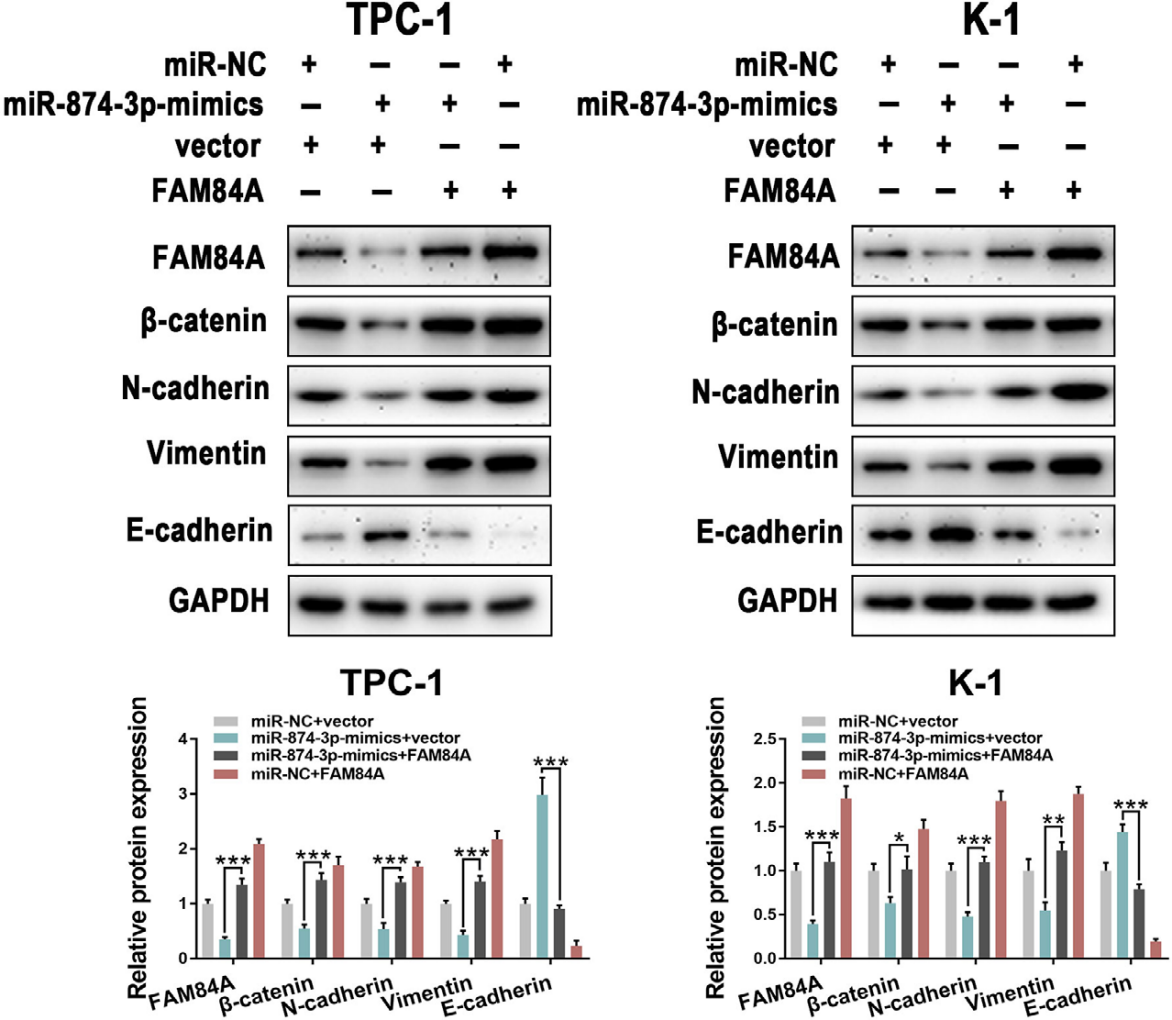
miR‐874‐3p regulates Wnt/β‐catenin signaling pathway and EMT by targeting FAM84A in PTC. (A) Upregulation of miR‐874‐3p suggested the increase in E‐cadherin but the decrease in N‐cadherin and vimentin, as well as β‐catenin. These effects could be partially recovered by cotransfecting with miR‐874‐3p‐mimics + FAM84A. Results were representative of three independent experiments and presented as the mean ± SD, two‐tailed *t*‐tests, *P < 0.05, **P < 0.01, and ***P < 0.001.

To investigate whether miR‐874‐3p involved in EMT and Wnt/β‐catenin signaling pathway by targeting FAM84A, expression of EMT‐related and β‐catenin protein was detected by western blot, followed by upregulation of miR‐874‐3p or FAM84A. As expected, overexpression of miR‐874‐3p induced the increased expression of E‐cadherin but the decreased expression of vimentin, N‐cadherin, and β‐catenin. By cotransfecting with miR‐874‐3p‐mimics and FAM84A vector, these effects could partially be recovered (Fig. 9A). These results further revealed that FAM84A was a downstream functional regulator of miR‐874‐3p involved in EMT and Wnt/β‐catenin signaling pathway.

## 4 Discussion

FAM84A is a collagen protein that is upregulated in several human tumors and may play a key role in the development of colon and liver cancer by promoting cell migration [9, 10]. In the current study, we first discovered that FAM84A was overexpressed in PTC according to TCGA and GEO databases, along with our own PTC tissues and cell lines. Furthermore, PTC patients with high expression of FAM84A tended to possess larger tumor size, higher lymph node metastasis rate, and advanced TNM stage. These results preliminarily suggested that FAM84A could be related to the development of PTC.

Given that FAM84A was upregulated in PTC, we used loss‐of‐function approaches to investigate the functional role of FAM84A in PTC cells. Knockdown of FAM84A in PTC cells resulted in the inhibition of cell proliferation, migration, and invasion, and cell cycle, while increased the apoptosis rate. Taken together, these results suggested that FAM84A acted as an oncogene on PTC carcinogenesis.

FAM84A was found localized in the subcytoplasmic membrane region and played a role in cellular migration [9]. However, the mechanism by which it works remains unclear. In the present study, results from GSEA showed that FAM84A expression was positively associated with epithelial–mesenchymal transition and Wnt/β‐catenin signaling pathway in PTC. The Wnt/β‐catenin pathway involved in the regulation of cell proliferation, polarity, and cell fate determination, which is a foundational mechanism during embryonic development and tissue homeostasis [24, 25]. It has been reported that overactivation of the Wnt/β‐catenin pathway induces tumorigenesis in the thyroid cancer [12, 26, 27]. Furthermore, Eduardo et al. reported that FAM84A was positively related to Wnt/β‐catenin signaling in rat hippocampal neurons [28]. β‐Catenin, when upregulated by upstream Wnt signaling, is translocated into the nucleus and transcribes various tumor‐promoting gene [14]. In the present study, a positive correlation was observed between FAM84A expression and β‐catenin expression by analyzing TCGA database. To further investigate the role of FAM84A in Wnt/β‐catenin signaling pathway, immunofluorescence and western blot analyses indicated that downregulation of FAM84A inhibited expression of β‐catenin in nucleus. Moreover, the TOP/FOP transcriptional activity was suppressed in PTC cells treated with si‐FAM84A. β‐Catenin also plays an important role in the EMT signaling [29]. The EMT process is usually activated in the process of tumor cell metastasis, which is the main pathological event of tumorigenesis [30]. Molecular changes during EMT have been reported in thyroid carcinogenesis [31, 32, 33], which is accompanied by the cell shape changed into spindle‐shaped fibroblast‐like, which means the loss of cell–cell contact and cell polarity [34]. E‐cadherin, N‐cadherin, and vimentin are identified as the key regulatory transcription factors for EMT, during which E‐cadherin governs the epithelial genes while N‐cadherin and vimentin activate the mesenchymal genes [35, 36]. In our study, we found that knockdown of FAM84A upregulated E‐cadherin and downregulated N‐cadherin, vimentin, and β‐catenin. Meanwhile, we discovered that TPC‐1 and K‐1 cells displayed an epithelial‐like shape, which suggested FAM84A may contribute to PTC carcinogenesis by promoting the EMT process. Taken together, what we found in this study revealed that FAM84A may activate the EMT and Wnt/β‐catenin signaling pathway in PTC.

Moreover, it is an interesting topic to explore the upstream target of FAM84A. After screening the public databases and conducting a series of verifications, we determined that miR‐874‐3p was a key regulator to FAM84A. Dysregulation of miR‐874‐3p has been reported to be implicated in the carcinogenesis and metastasis of several malignancies, such as osteosarcoma, breast cancer, gastric cancer, head and neck squamous cell carcinoma, and colorectal cancer [37, 38, 39, 40, 41]. Besides, miR‐874‐3p and ITGB4, as a potential miRNA‐mRNA regulation, have been discovered characteristic for PTC samples [42]. In this study, we observed that FAM84A was negatively regulated by miR‐874‐3p and played an oncogenic role through Wnt/β‐catenin signaling in PTC. At present, miR‐874‐3p and FAM84A regulation has never been studied as a complete signal axis in other tumors, but we found that this regulation existed in PTC, so it also may be distinctive.

## 5 Conclusions

In summary, this study highlighted the oncogenic role of FAM84A in PTC by promoting the EMT process and activating the Wnt/β‐catenin signaling pathway. Moreover, miR‐874‐3p was identified that could target the 3’UTR of FAM84A to suppress its expression. The results what we found suggested that FAM84A may be a potential biomarker and a useful therapeutic target of PTC.

## Conflict of interest

The authors declare that they have no conflict of interest.

## Author contributions

MS designed the experiments. YD gave the first idea, performed the most experiments, and was a major contributor in writing the manuscript. LW performed part of the experiments. XZ, JC, and HT analyzed the data with assistance from YS, HZ, and XW; All authors read and approved the final manuscript.

### Peer Review

The peer review history for this article is available at https://publons.com/publon/10.1002/1878‐0261.12941.

## Supporting information


**Fig S1.** IHC analysis of FAM84A protein.

**Fig S2.** Morphological change of TPC‐1 and K‐1 cells, TOP‐flash/FOP‐flash luciferase reporter assay.

**Fig S3.** Immunofluorescence and western blot analysis of FAM84A and β‐catenin in TPC‐1 cells.

**Fig S4.** RNA immunoprecipitation assay and RNA pull‐down assay verify the interaction between miR‐874‐3p and FAM84A.

**Fig S5.** Expression of miR‐874‐3p in PTC tissues and cell lines, validation of transfection efficiency.

**Fig S6.** Edu assay and wound‐healing assay.

**Table S1.** Correlation between FAM84A expression and clinicopathological characteristics of PTC patients.

Supplementary Material
