# Supplementary figures and images for: The direct miR‐874‐3p‐target FAM84A promotes tumor development in papillary thyroid cancer
Source: Mol Oncol. 2021 Mar 23;15(5):1597–614. doi: 10.1002/1878-0261.12941 (PMC8096794; doi:10.1002/1878-0261.12941)

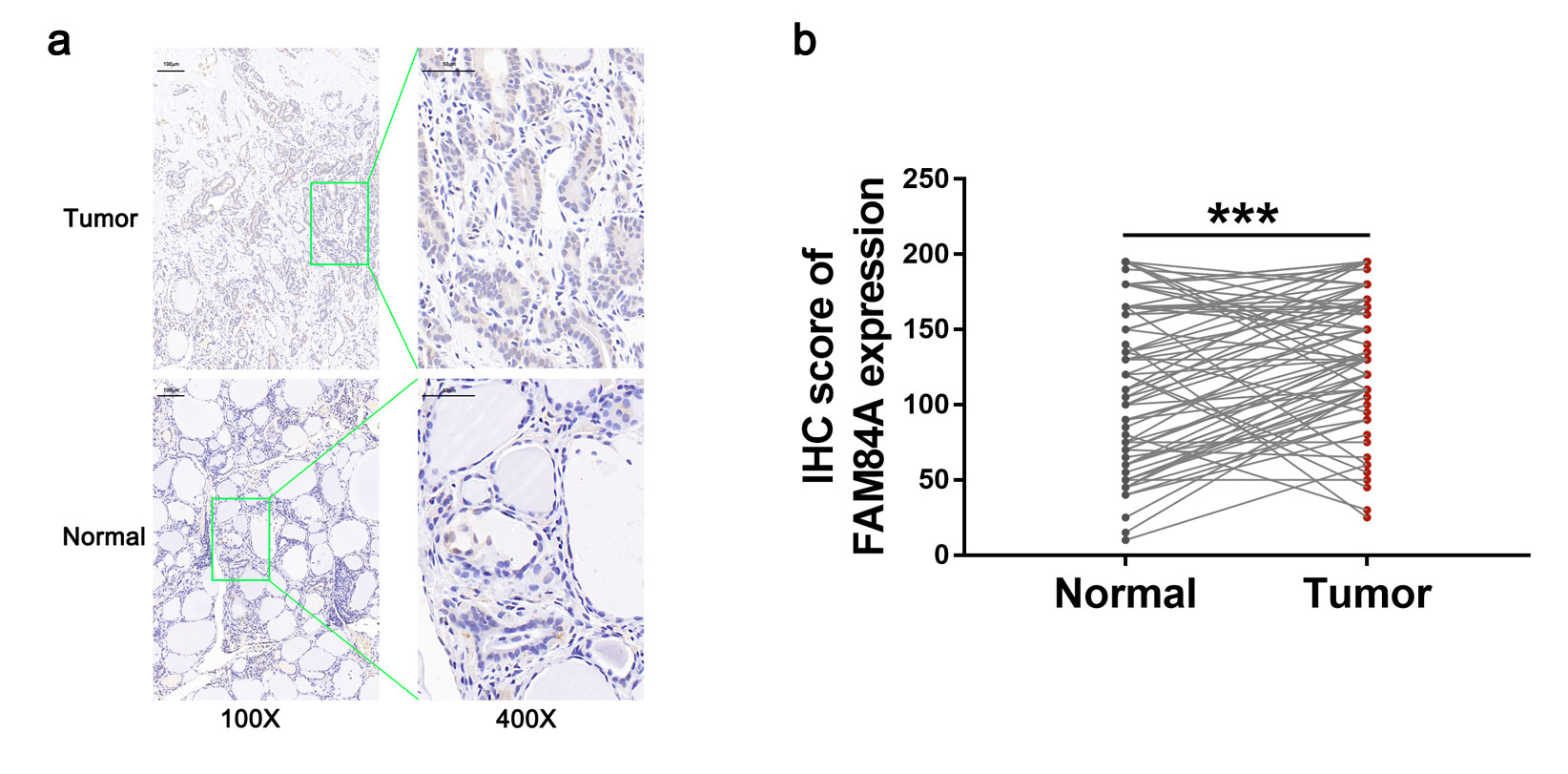

Supplement: Supplementary file 1 — Fig S1. IHC analysis of FAM84A protein. [file MOL2-15-1597-s005.jpg]

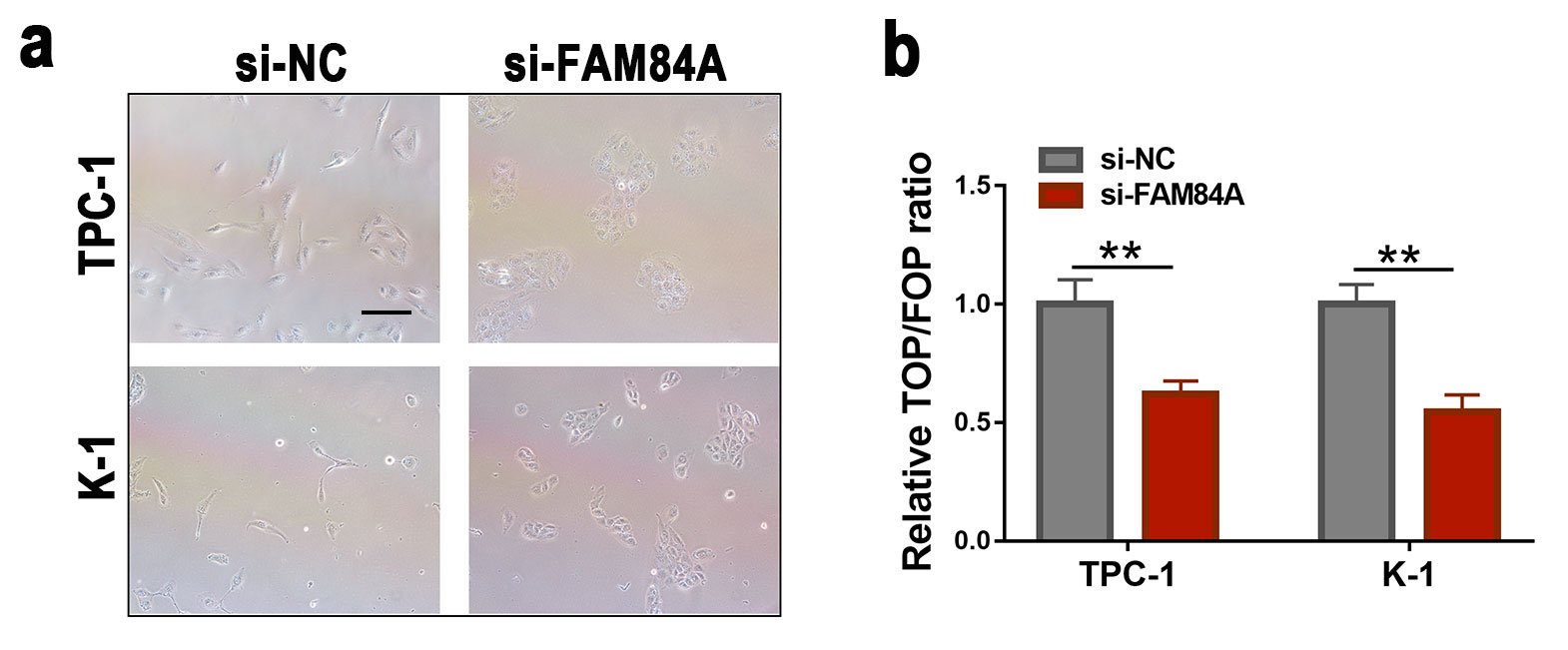

Supplement: Supplementary file 2 — Fig S2. Morphological change of TPC‐1 and K‐1 cells, TOP‐flash/FOP‐flash luciferase reporter assay. [file MOL2-15-1597-s006.jpg]

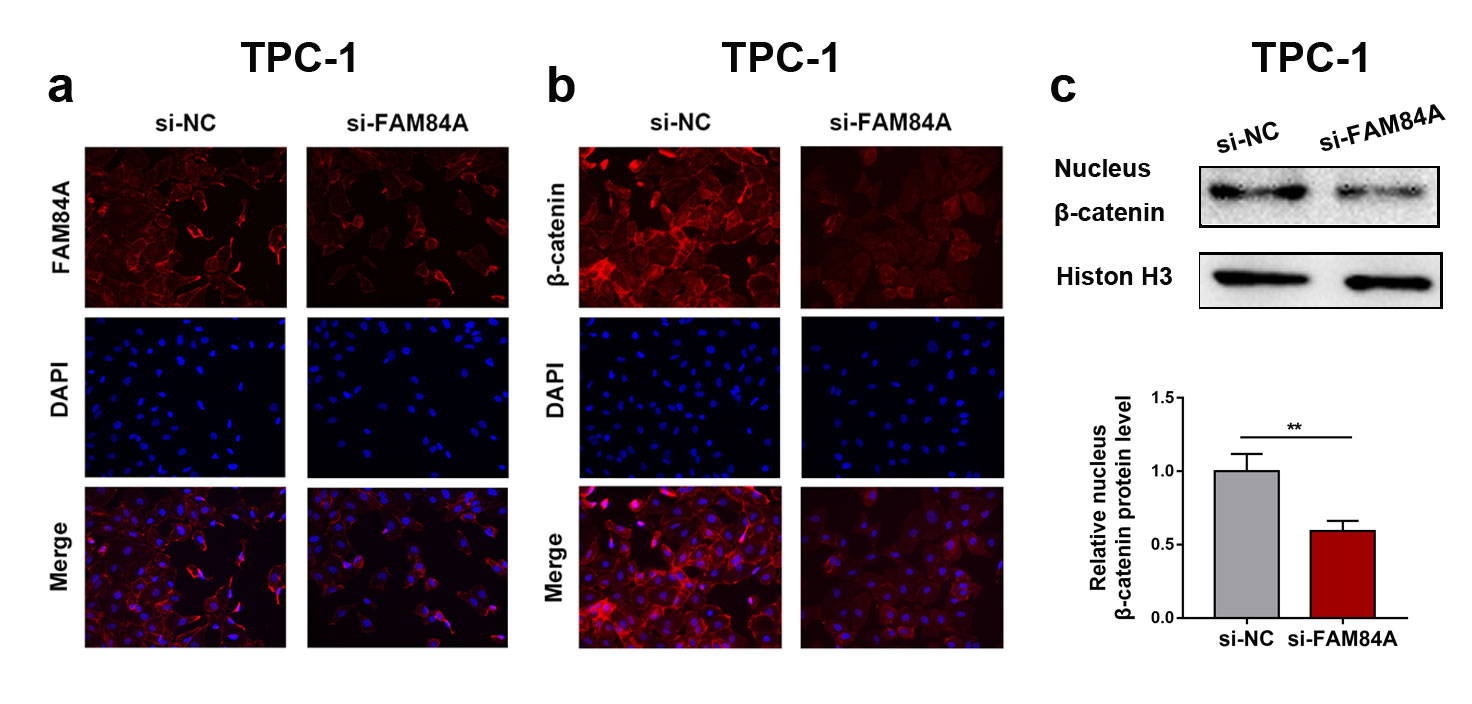

Supplement: Supplementary file 3 — Fig S3. Immunofluorescence and western blot analysis of FAM84A and β‐catenin in TPC‐1 cells. [file MOL2-15-1597-s007.jpg]

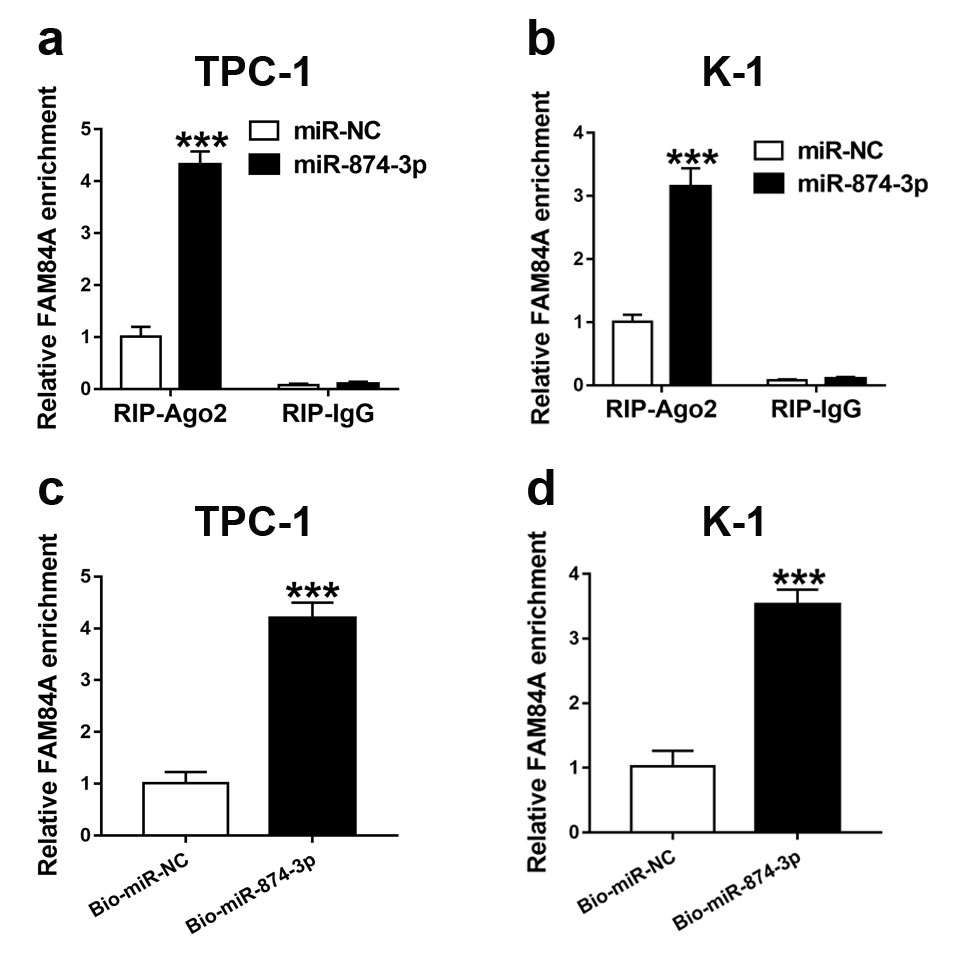

Supplement: Supplementary file 4 — Fig S4. RNA immunoprecipitation assay and RNA pull‐down assay verify the interaction between miR‐874‐3p and FAM84A. [file MOL2-15-1597-s004.jpg]

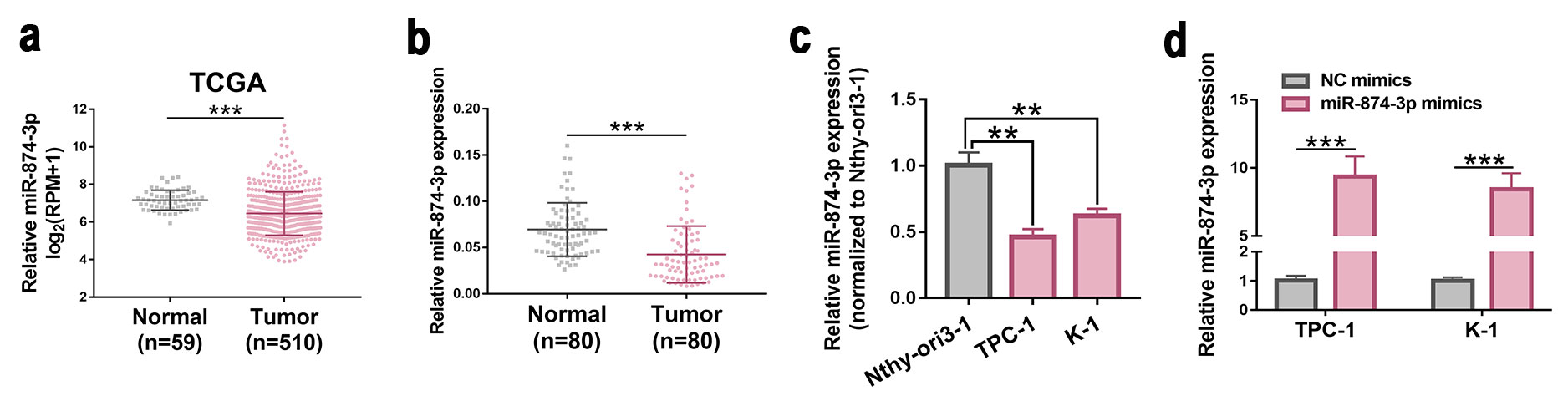

Supplement: Supplementary file 5 — Fig S5. Expression of miR‐874‐3p in PTC tissues and cell lines, validation of transfection efficiency. [file MOL2-15-1597-s002.jpg]

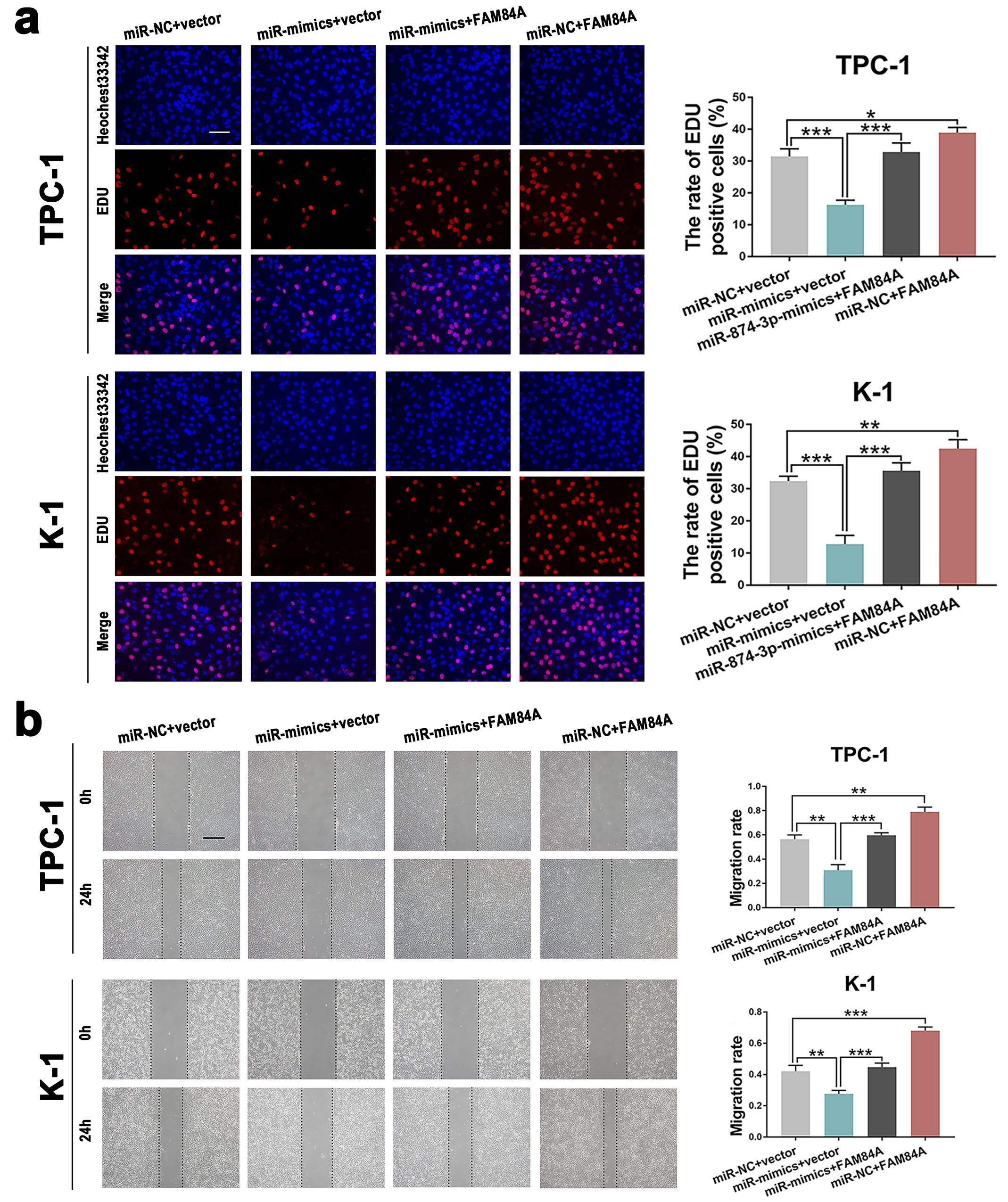

Supplement: Supplementary file 6 — Fig S6. Edu assay and wound‐healing assay. [file MOL2-15-1597-s003.jpg]
