# Supplementary material for: The direct miR‐874‐3p‐target FAM84A promotes tumor development in papillary thyroid cancer
Source: Mol Oncol. 2021 Mar 23;15(5):1597–614. doi: 10.1002/1878-0261.12941 (PMC8096794; doi:10.1002/1878-0261.12941)
Supplement: Supplementary file 7 — Table S1. Correlation between FAM84A expression and clinicopathological characteristics of PTC patients. [file MOL2-15-1597-s001.docx]

**Supplementary Table 1. Correlation between FAM84A expression and clinicopathological characteristics of PTC patients**

| **Characteristics** | **Number** | **FAM84A expression** | | **P value^#^** |
| --- | --- | --- | --- | --- |
|  |  | **High** | **Low** |  |
| Gender | | | | |
| Male | 21 | 7 | 14 | 0.075 |
| Female | 59 | 33 | 26 |  |
| Age (years) | | | | |
| ≤55 | 46 | 21 | 25 | 0.366 |
| >55 | 34 | 19 | 15 |  |
| Multifocality | | | | |
| Yes | 16 | 10 | 6 | 0.264 |
| No | 64 | 30 | 34 |  |
| Extral thyroidal extension | | | | |
| Yes | 21 | 13 | 8 | 0.204 |
| No | 59 | 27 | 32 |  |
| Tumor size (cm) | | | | |
| ≤2 | 65 | 29 | 36 | **0.045*** |
| >2 | 15 | 11 | 4 |  |
| Lymph node metastasis | | | | |
| Yes | 38 | 24 | 14 | **0.025*** |
| No | 42 | 16 | 26 |  |
| TNM stage | | | | |
| I + II | 55 | 23 | 32 | **0.030*** |
| III + IV | 25 | 17 | 8 |  |

**^#^**, Chi-square detection; *****, P< 0.05 was considered significant.

The significant results are in bold.
