# Supplementary material for: The direct miR‐874‐3p‐target FAM84A promotes tumor development in papillary thyroid cancer
Source: Mol Oncol. 2021 Mar 23;15(5):1597–614. doi: 10.1002/1878-0261.12941 (PMC8096794; doi:10.1002/1878-0261.12941)
Supplement: Supplementary file 8 — Supplementary Material [file MOL2-15-1597-s008.docx]

**The direct miR-874-3p-target FAM84A promotes tumor development in papillary thyroid cancer**

Yu Ding^1*^, Luyao Wu^1*^, Xi Zhuang^1*^, Jingsheng Cai^1^, Houchao Tong^1^, Yan Si^1^, Hao Zhang^1^, Xiaoting Wang^1^, Meiping Shen^1^

^1^ Department of General Surgery, the First Affiliated Hospital of Nanjing Medical University, Nanjing, Jiangsu Province, China

**Supplementary Figures**

**Supplementary Figure 1**. IHC analysis of FAM84A protein

**a** Immunohistochemistry staining was used to determine the protein level of FAM84A in PTC tissues and none-PTC tissues. Original magnification 100 × and 400 ×. **b** The protein level of FAM84A was significantly increased in human PTC samples. Results were representative of three independent experiments and presented as the mean ± SD, Paired t test, ****p* < 0.001.

**Supplementary Figure 2**. Morphological change of TPC-1 and K-1 cells, TOP-flash/FOP-flash luciferase reporter assay

**a** TPC-1 and K-1 cells displayed a remarkable morphological change from the typical spindle-like shape to an epithelial-like shape after knockdown of FAM84A. Scale bar = 200μm. **b** TOP/FOP transcriptional activity was remarkably inhibited in PTC cells treated with si-FAM84A compared with their negative control groups. Results were representative of three independent experiments and presented as the mean ± SD, Two-tailed t tests, ***p* < 0.01.

**Supplementary Figure 3.** Immunofluorescence and western blot analysis of FAM84A and β-catenin in TPC-1 cells

**a, b** Immunofluorescence analysis indicated that FAM84A was mainly located in cytoplasm, β-catenin was located both in cytoplasm and nuclei, and that downregulation of FAM84A inhibited expression of nuclear β-catenin. Scale bar = 200μm. **c** Western blot further revealed that downregulation of FAM84A inhibited the protein expression of nucleus β-catenin. Results were representative of three independent experiments and presented as the mean ± SD, Two-tailed t tests, ***p* < 0.01.

**Supplementary Figure 4**. RNA immunoprecipitation assay and RNA pull-down assay verify the interaction between miR-874-3p and FAM84A

**a, b** RIP assay evaluated the enrichment of FAM84A mRNA in K-1 and TPC-1 cells transfected with miR-874-3p mimic or miR-NC. **c, d** RNA Pull-down assay was used to confirm that FAM84A is a target of miR-874-3p in PTC. Results were representative of three independent experiments and presented as the mean ± SD, Two-tailed t tests, ****p* < 0.001.

**Supplementary Figure 5.** Expression of miR-874-3p in PTC tissues and cell lines, validation of transfection efficiency

**a, b** According to data from TCGA database and our own 80 paired PTC tissues, miR-874-3p was found downregulated in PTC. **c** miR-874-3p was also downregulated in TPC-1 and K-1 cells. **d** TPC-1 and K-1 cells were transfected with miR-874-3p mimics and their negative control groups, the efficiency of transfection was verified by qRT-PCR. Results were representative of three independent experiments and presented as the mean ± SD, Two-tailed t tests, ***p* < 0.01, ****p* < 0.001.

**Supplementary Figure 6.** Edu assay and wound-healing assay

**a, b** Edu assay and wound-healing assay displayed that overexpression of miR-874-3p inhibited proliferation and migration of PTC cells, which could be partially reversed by upregulation of FAM84A. For Edu assay, scale bar = 200μm. For wound-healing assay, scale bar = 100μm. Results were representative of three independent experiments and presented as the mean ± SD, Two-tailed t tests, **p* < 0.05, ***p* < 0.01, ****p* < 0.001.
